# Investigating and Managing the Impact of Using Untreated Wastewater for Irrigation on the Groundwater Quality in Arid and Semi-Arid Regions

**DOI:** 10.3390/ijerph18147485

**Published:** 2021-07-13

**Authors:** Hany F. Abd-Elhamid, Shaimaa M. Abd-Elmoneem, Gamal M. Abdelaal, Martina Zeleňáková, Zuzana Vranayova, Ismail Abd-Elaty

**Affiliations:** 1 Water and Water Structures Engineering Department, Faculty of Engineering, Zagazig University, Zagazig 44519, Egypt; hany_farhat2003@yahoo.com (H.F.A.-E.); drgamal_abdelaal@yahoo.com (G.M.A.); Eng_abdelaty2006@yahoo.com (I.A.-E.); 2 Center for Research and Innovation in Construction, Faculty of Civil Engineering, Technical University of Košice, 04200 Košice, Slovakia; 3 Belbis High Institute for Engineering, Belbes, El Sharqia 44519, Egypt; shaimaamohamed358@yahoo.com; 4 Institute of Environmental Engineering, Faculty of Civil Engineering, Technical University of Košice, 04200 Košice, Slovakia; 5 Department of Building Facilities, Faculty of Civil Engineering, Technical University of Košice, 04200 Košice, Slovakia; zuzana.vranayova@tuke.sk

**Keywords:** wastewater, treatment, irrigation, groundwater quality, COD

## Abstract

This study aims to investigate the impact of using untreated wastewater in irrigation. Different scenarios of management were applied by mixing it with treated wastewater or freshwater on groundwater quality. A hypothetical case study is presented. The numerical model of MODFLOW is used in the simulation by applying four stages (21 scenarios) including: different values of pumping rates, changing wastewater recharge rates, and a combination of the previous scenarios. Additionally, protection scenario for groundwater was applied by using different values of mixing of freshwater with wastewater. The simulation was carried out for the contamination of Chemical Oxygen Demand COD and the concentration reached 48.6 ppm at a depth of 25 m and 19.41 ppm at a depth of 50 m in the base case. The results showed a negative impact on groundwater quality had occurred due to increasing the pumping rates, wastewater recharge rates, and combination between two scenarios, which led to an increase of the contaminants in the aquifers. However, positive protection effects occurred due to mixing the wastewater with treated wastewater. The results of COD concentration in groundwater using treated wastewater reached 81.82, 77.88, 74.03, 70.12, and 66.15 ppm at a depth of 25 m and 53.53, 50.95, 48.43, 45.87, and 43.28 ppm at a depth of 50 m, at concentrations of 93, 88.52, 84.14, 79.7, and 75.19 ppm with constant pumping and recharge rates of 4320 m^3^/d and 547.5 mm/year, respectively. The using of treated wastewater could improve the groundwater quality to be used in the irrigation process and help to minimize groundwater contamination. Moreover, the abstraction of the groundwater should be optimized, and the qualities of wastewater should be constrained in agriculture to protect the groundwater quality.

## 1. Introduction

Water scarcity is considered the major challenge that faces many countries around the world [1]. It is an imbalance condition that occurs because of a lack of freshwater resources and increasing water demand. The amount of water on the surface of the planet is estimated at 1388 million cubic kilometers, about 97% of that amount is in the form of salt water, and only 3% is in the form of freshwater. The largest proportion of this freshwater, between 48 and 69%, is found in the form of permanent ice caps in the Antarctic, ice in mountainous regions, and the Arctic. Groundwater accounts for nearly 30% of freshwater and less than 1% of freshwater distributed in the form of lakes and rivers on the surface of the earth. This is evidence that about less than 1% of the total available water can be easily accessed to meet different needs [2,3].

Water shortage imposes severe conditions on both the safety of the society and development of the economy. The agricultural sector is the most water-consuming sector because it consumes about 80% of the total water used, so it is the sector most affected by a water shortage, followed by the domestic sector, as both of them witness increased consumption over the years. This is due to the increase in population and changes in temperature, especially its rise and the demands of the standard of living [4]. The continuing pressure on freshwater resources is due to global climate change; it has an adverse influence on water scarcity, particularly in arid regions with heavily populated areas [2]. The global population growth is approximately 83 million per year, or 1.15%, as the current population is estimated at 7.6 billion, and by 2035 it is expected to reach 8.6 billion. The United Nations in 2017 estimated that by 2055 the rate of increase will reach 30%, which would be about 9.8 billion and by 2100 it will be 11.2 billion [5]. In the year 2050, global water use is expected to increase by 55%. This is due to the increase in population and increasing consumption of manufacturing and households. The reports indicated that the municipal sector consumes about 11% of the global water and that the agricultural sector is the main consumer of water in the world. It has also been observed that about 88% of the water used in the household sector is discharged as wastewater, i.e., about 330 km^3^ annually. The quantity that used to irrigate approximately 40 million hectares of the land area and represents about 15% of the total cultivated land area [6]. The water need is estimated at the present time about 4600 km^3^ per year for all uses and by 2050, the percentage of increase is 20 to 30%, in order to reach 5500 to 6000 km^3^ annually. Globally, by 2025 the demand for water for agricultural purposes will increase by 60% and the percentage of water used for industrial purposes is about 20% and 10% of the total for domestic purposes. In 2010 it was estimated that the amount of global groundwater used annually is about 800 km^3^, and by 2050 probably that the increase in withdrawal will reach 1100 km^3^ per year, or about 39% [7]. In addition to the natural resources, the desalination and the reuse of water are the addition methods to increase the amount of freshwater resources [2]. Groundwater is the available source of freshwater in arid and semi-arid regions such as the countries of the Mediterranean, especially North Africa. These areas are suffering from a rapid increase in population, pollution, and excessive consumption of groundwater, which leads to a severe shortage of the water resources needed. This is increasing the attention for water quality assessments to solve major issues of aquifer problems in these areas, especially coastal areas such as overconsumption, pollution, and salinization and to provide optimal support for the protection of water resources in the long term [8]. Surface water resources are very limited in arid regions, such as Egypt. Precipitation is rare in these areas with the high rate of evaporation and the decrease in recharge. The constraints resulting from the poverty and poor quality of groundwater in arid regions confront the use and management of groundwater and lead to obstacles in regional and economic development [9].

The wastewater contributes with partial assistance to compensate for the water shortage. Nearly 40% of the world’s population suffers from the lack of water needed for irrigation, which causes agricultural losses. As a result of the water shortage, especially in arid and semi-arid regions, wastewater is used as the main water source, and this is due to its large quantity, as wastewater discharge reaches 400 billion cubic meters/year globally. The concentration of wastewater varies from raw to dilute due to the different disposal outlets. For example, urban wastewater from domestic, commercial, and industrial drainage; the surface runoff of rainwater; treated wastewater through treatment plants; and finally reclaimed or recycled water. Sewage water is considered a stable source of water because it is not dependent on climatic conditions or rain, as it is easy to use for agriculture throughout the year. In addition, it contains nutrients that help crops grow and in turn reduces fertilizer costs and the use of chemicals [10]. Wastewater is usually composed of 99% water and 1% suspended, colloidal, and dissolved solids. Organic matter, suspended solids, nutrients (primarily nitrogen and phosphorus), heavy metals, new toxins (antibiotics, chemicals, care products, pesticides, phenolic compounds, antibiotic resistant bacteria, volatile organic compounds, polycyclic aromatic hydrocarbons, and genes), and pathogens are all believed to be found in wastewater, depending on its source (protozoans, parasitic worms, bacteria, and viruses). Although there are numerous advantages of using wastewater in agriculture, there are also numerous risks to this method, including various pathogens in farmers and users of food products irrigated by wastewater; deposition of heavy metals, salts, growth hormones, antibiotics, and other harmful substances in the soil; poor hydraulic conductivity due to soil pores clamping with suspended wastewater solids; and reduced quality of farm crops because toxins transported from wastewater to soil are accumulated [11].

As a result of the increase in the population and the economy growth a shortage of resources and an increase in pollution, so about 12% of the world’s population uses unsuitable water from sources that are not safe. About 30% of the population in the world, or nearly 4 billion people, live without using any methods for sanitation operations, so in developing countries they dispose of nearly 90% of wastewater into untreated water. Among the causes of pollution is that 730 million tons of wastewater is disposed annually with other liquid wastes into the water. As for industrial waste, it is estimated at about 300 to 400 megatons per year. Despite the risk of using wastewater, it has become a common and important issue for livelihoods, as it is recycled in agriculture [7].

Massive amounts of wastewater are generated daily in homes, factories, and agriculture. Wastewater accounts for 50–80% of residential household water consumption with global wastewater discharge valued at 400 billion m^3^/year, contaminating nearly 5500 billion m^3^ of water annually [11]. The random disposal of human, industrial, and sewage waste affects the physical and chemical properties of rivers. Therefore, it was mainly established by standards of water quality to improve the current situation and suitability of the methods of use. On this basis, any change in the chemical, physical, and biological properties lead to a deterioration in the quality of water, its pollution, and the imbalance of the ecosystem. Water quality includes a system of variables such as temperature, pH, oxygen concentration, etc. and for testing the quality of water before using it is very important for drinking purposes or for industrial, agricultural, domestic, or commercial purposes. Therefore, the World Health Organization (WHO) and the Food and Agriculture Organization (FAO) have set standard limits for pollutants in irrigation and drinking water [12]. The cumulative amount of oxygen needed to fully oxidize all organic matter to H_2_O and CO_2_ is known as the Chemical Oxygen Demand (COD). The values of the chemical oxygen demand rise due to discharges from the drain polluted with sewage water and agricultural wastes, and also because of the high load of organic matter and the lack of water’s ability to self-purification. Biochemical Oxygen Demand (BOD) can be defined as the amount of Dissolved Oxygen (DO) that is consumed in order for the decomposition process of organic matter to take place by the microorganisms in the water [13].

The guidelines for COD according to (WHO 2006) for drinking water is 10 mg/L, according to Egyptian Drinking Water Quality Standards (2007) is 10 mg/L and for BOD is 3 mg/L [14]. As an additional water supply, the re-use of wastewater for irrigation is attracting international attention. The reuse of agricultural wastewater can be categorized as direct and indirect re-use of wastewater. Direct reuse of wastewater means the method where the water from the wastewater treatment system is provided directly, while indirect re-use of wastewater is a method where wastes from wastewater treatment plant (WWTP) or untreated wastewater is obtained downstream. The irrigation water quality is based on the quality of the WWTP effluent when the wastewater reused directly. For indirect case the rapid industrialization has led to an increase in number of WWTPs, which has intensified the impacts of indirect wastewater reuse on irrigation water [15].

Water reuses and recycles for agriculture and drinking water through surface and groundwater sources is a traditional and long-standing practice. The WHO now recommends a system that integrates elements of risk assessment and risk control to ensure water quality for agricultural reuse [16]. The protection of wastewater reuse is a critical issue for crop irrigation around the world. If wastewater is not properly treated until being used for irrigation, it can damage the soil (salinization, toxicity from sodium, boron ions, and chloride; decreased aeration and pore clogging from suspended solids in wastewater; structural deterioration; and reduced hydraulic conductivity), as well as agricultural production (excess nutrients cause heavy metal accumulation, biological load, and delayed or erratic growth of the plant), in relation to groundwater (by seeping of unnecessary nitrates). Where practicable, treated wastewater should be reused, and drainage methods should mitigate the potential negative impacts on the atmosphere and public health. The most appropriate treatment system for drainage before it is used as irrigation water is one that produces an effluent that satisfies quality criteria from a microbiological and chemical standpoint while requiring minimal operation and maintenance [11].

A number of studies indicated that activated sludge effluents would require extra treatment in order to reduce the human health risk of agricultural water reuse. Contreras et al. (2017) [17] performed a cross-sectional database analysis on the threat of diarrhea and wastewater pollution in Mexico. The aim of the analysis was to include an updated estimate of the health risk and to update the 2006 World Health Organization (WHO) recommendations for wastewater reuse. The results indicated that people who were exposed to wastewater had a higher risk of diarrhea than those who were not [18]. Abd-Elhamid et al. [19] evaluated the effect of different pumping schemes on the groundwater quality due to the seepage from open polluted drains using VISUAL MODFLOW with different depths, locations, and rates the result illustrated that the groundwater contamination is very sensitive to over pumping and the pumping schemes should be optimized. The benefits are the recharge of underground reservoirs substitute the lack of freshwater, the utilization of nutrients contained in the wastewater. This leads to a decrease in the use of fertilizers and the treatment of the groundwater aquifer in the soil. This results in a lack of direct discharge, and supply of nutrients to water bodies [20]. Abd-Elaty et al. [21] carried out geotechnical and numerical study for applied solutions to reduce soil and groundwater contamination by fertilizers in arid and semi-arid regions in the Eastern Nile Delta, Egypt. The result showed that silty clay soils are able to contain the contaminations and preserve the groundwater quality, and, more than pumping, has a positive effect for groundwater contamination and negative effect for soil pollution.

As for the economic features, the following were used: reduced treatment costs for wastewater, reduced fertilizer costs, and reduced costs of freshwater; as for the negative aspects, they are formed in the damage to human health as well as environmental damage, as it requires a special distribution system for wastewater in order to be stored in the seasons in which it is not used [20].

Abd-Elhamid et al. [22] developed a numerical model to evaluate efficiency of lining materials for open polluted drains including clay, bentonite, geomembranes, and concrete to reach 43, 89.6, 91.4, and 93% compared with the base case. Additionally, the result showed that the geomembranes high performance for groundwater protection compare with these materials due to high durability and low cost. Abd-Elaty et al. [23] developed a numerical study for groundwater protection using new process techniques in the Nile delta, Egypt, including the boundary conditions of canals and polluted drains, the location of the polluted drain, installing a cut-off wall in the polluted drain sides, and lining materials. Increasing the aquifer boundary conditions for canals, decreasing the head of polluted drain, decreasing the top aquifer conductivity, and lining of the polluted drain section can minimize the aquifer contamination while installing the cut off has a good effect in shallow aquifers with no effect in the deep aquifer. Treated wastewater is commonly used in the world, so it is suitable for irrigation. According to previous studies, more than 10% of the global population consumed agricultural crops grown through irrigation with wastewater [15]. In order to enhance the use of remaining natural resources of freshwater, water management, developed catchment, distribution, storage, and appropriate infrastructure for water is essential [2].

In this study a numerical model MODFLOW is used to investigate the effect of using untreated wastewater on groundwater quality considering different scenarios of abstraction rates, aquifer recharge and mixing wastewater with freshwater, which improve the water quality and protect groundwater aquifers contamination. Additionally, the study produces good tools to improve the groundwater quality in arid and semi-arid regions, which have shortage in water resources using different percentage of untreated wastewater with freshwater.

## 2. Materials and Methods

### 2.1. Study Area Description and Flow Domain

**Figure 1:**
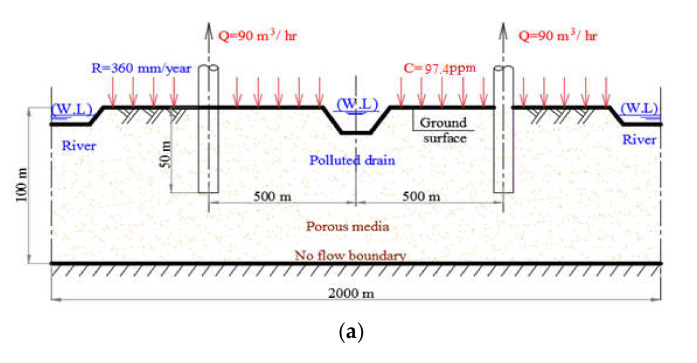

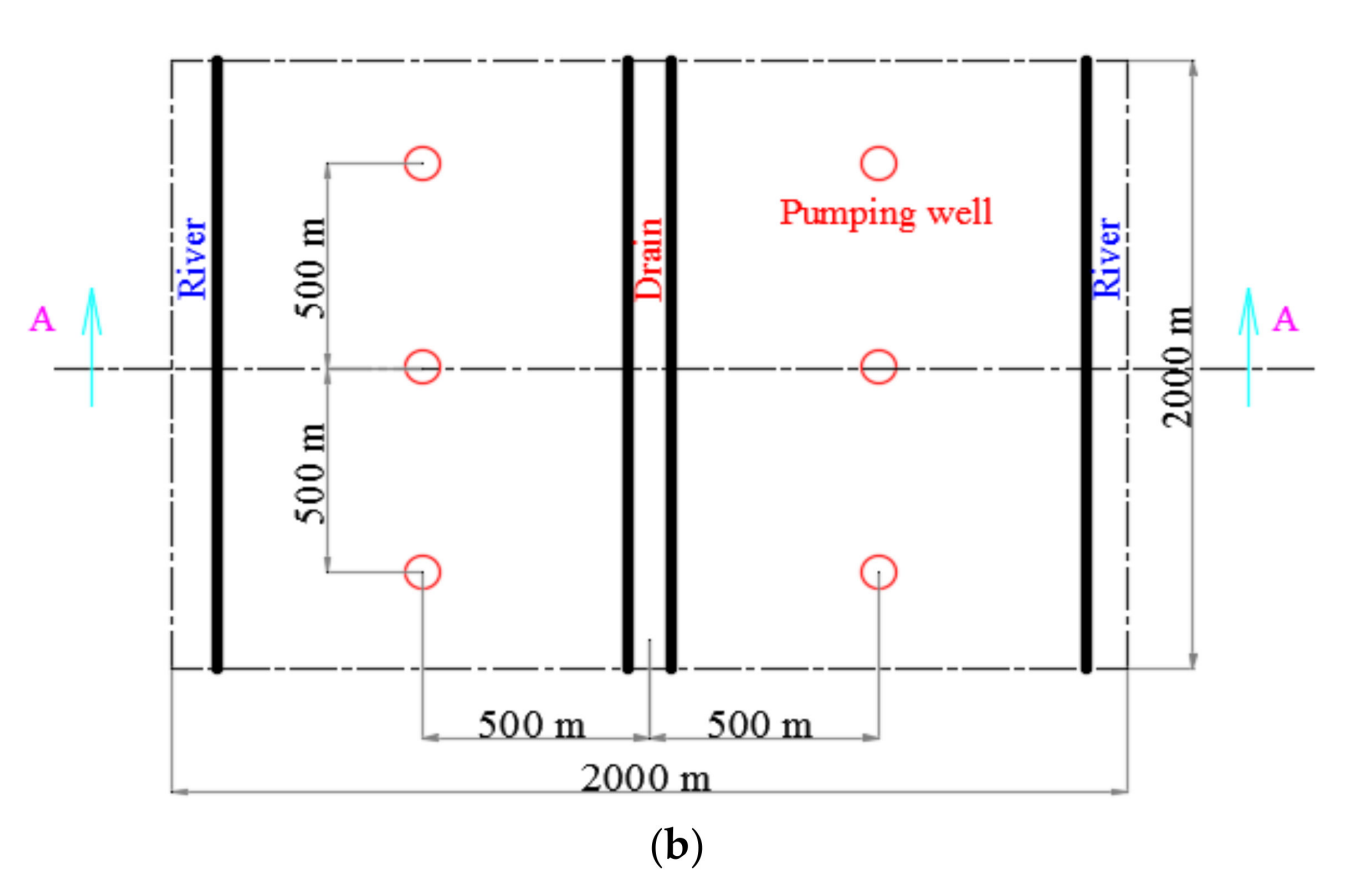
Cross section and plan of the study area (**a**) section elevation A-A and (**b**) plan.

The study area is simulated using a hypothetical case with total area of 4 km^2^ with length and width equals to 2000 m while the depth is 100 m. It is divided into cells where the area of a cell is 400 m^2^, also the domain is divided into 100 columns, 100 rows, and 20 layers. The case study is assigned by main drain, which allocated in the middle and two canals that exist parallel at the boundaries of the model; each of them is located 1000 m from the main drain. Furthermore, in the middle of the distance between the drain and the canal there are six wells distributed on each side at a distance of 500 m from drain center (See Figure 1). The homogeneity of natural formation is used for simplification of real aquifers and the heterogeneity plays an important role in the flow and transport process. Additionally, if the transfer time ranges are compared to system change and are relatively short the similar techniques can be used in the contaminant transport model. Generally, heterogeneity refers some sort of “preferential flow”, and it is important to consider when designing monitoring systems that determining preferential flows in heterogeneous systems is the most important consideration [24].

### 2.2. Analatical Solution

The calculation of groundwater head between the river and the drain used the following mathematical equation based on Darcy’s law [25]:

(1) $$K\left(h^{2}-h_{0}^{2}\right)-NX\left(L-x\right)+\frac{KX}{L\left(h_{0}^{2}-h_{l}^{2}\right)}=0$$

where: *h*: elevation of water (*L*) between the river and the drain, *h*_0_: the drain elevation located on the west (*L*), *h_l_*: the river elevation located on the east (*L*), *K*: the hydraulic conductivity (LT^−1^), *N*: is the porosity, and *X*: the distance along the aquifer from the drain located on the west (*L*).

**Figure 2:**
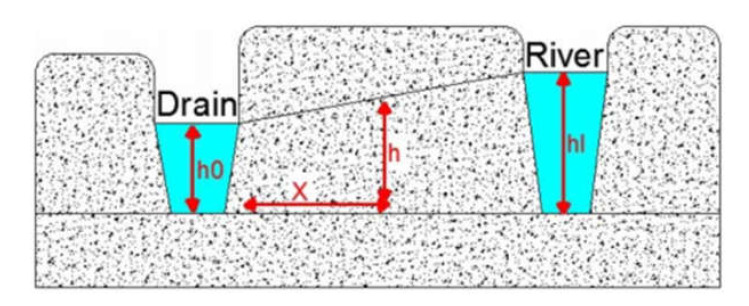
Parameters of numerical equation.

The analytical equation is based on the head between the drain and the canal, shown in Figure 2.

### 2.3. Numerical Model

The use of numerical models is useful to identify the groundwater flow and solute transport in aquifers. MODFLOW is used to evaluate groundwater quality and quantity for using untreated wastewater in irrigation. The governing equation for groundwater flow can be defined as following [26]:

(2) $$\frac{\partial }{\partial x}\left(K_{xx}\frac{\partial h}{\partial x}\right)+\frac{\partial }{\partial y}\left(K_{yy}\frac{\partial h}{\partial y}\right)+\frac{\partial }{\partial z}\left(K_{zz}\frac{\partial h}{\partial z}\right)+W=S_{S}\frac{\partial h}{\partial t}$$

The solute transport model was simulated using 3-D of MT3D model and the governing equation can be written as following [27]:

(3) $$\frac{\partial c}{\partial t}=\frac{\partial }{\partial x_{i}}\left(D_{ij}\frac{\partial c}{\partial x_{j}}\right)-\frac{\partial }{\partial x_{i}}\left(V_{i}C\right)+\frac{q_{S}}{\theta }C_{S}+\sum_{K-1}^{N}R_{K}$$

where: *K_xx_*, *K_yy_*, and *K_zz_*: the hydraulic conductivity along the *x*, *y*, and *z* coordinate axes (T^−1^), *h*: the potentiometric head (L), *W*: volumetric flux per unit volume representing sources and/or sink of water in (T^−1^), *S_S_*: specific storage of the porous material (L^−1^), *t*: time (T), *C*: groundwater concentration (ML^−3^), *D_ij_*: dispersion coefficient (L^2^T^−1^), *V*: seepage velocity (LT^−1^), *q_S_*: water flux of sources (positive) and sinks (negative) (T^−1^), *C_S_*: sources or sinks concentration (ML^−3^), *θ*: media porosity (dimensionless), and *R_K_*: chemical reaction term (ML^−3^T^−1^).

#### 2.3.1. Boundary Conditions

The boundary conditions of the model play a critical role for groundwater flow and solute transport [24]. In finite domains the results of both velocity and solute concentration are affected by imposed constraints also far from the boundaries. For instance, in trending media ignoring the influence of the boundary conditions may give erroneous conclusions on estimation of the transport process. The results of flow and solute transport in finite domains may be not consistent with those obtained for an unbounded domain and differences vary also with the different imposed constraints [28].

The water levels of the main drain are assigned below the ground surface (G.S) with range 2.5–2.8 m. The two rivers were allocated with average water levels ranges between 0.5–0.8 m and below the G.S and the bed levels between 3–3.30 m below G.S. In the first layer of the model the rate of annual recharge is assigned to 365 mm/year, while a contaminant for untreated wastewater recharge of Chemical Oxygen Demand (COD) is used in agricultural irrigation with a constant concentration equals to 97.4 mg/L [29].

#### 2.3.2. Hydraulic Parameters

The previous studies and mathematical calculations are used in allocating the hydraulic parameters to the study area including hydraulic conductivity in horizontal and vertical directions (*K_x_*, *K_y_*, *K_z_*), specific storage (*S_s_*), specific yield (*S_y_*), effective porosity (*n_eff_*), total porosity (*n_tota_*_l_). The hydraulic parameters of the case study are presented in Table 1.

#### 2.3.3. Model Calibration

MODFLOW is used to simulate the groundwater flow and contamination in the study area.

**Figure 3:**
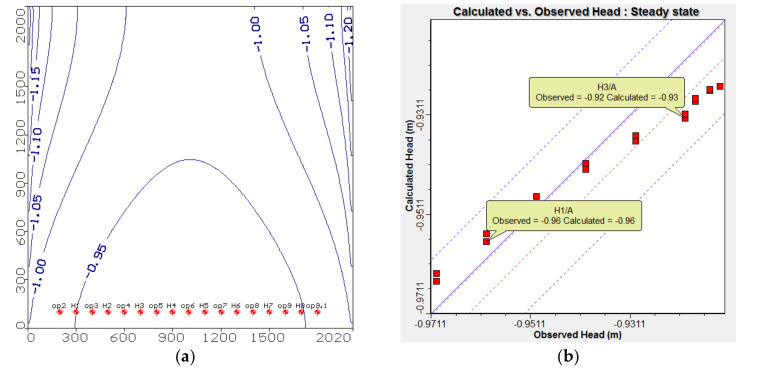
Head contour and model calibration.

The heads of the groundwater calculated by the numerical model are presented in Figure 3a, and the result compared with Equation (2) for calibration of groundwater heads. The results of calibration are shown a good match between the two models as presented in Figure 3b.

The number of observation wells is 16 and examined in the current calibration where the residual ranges between (−0.0012 to −0.001 m) with mean equals to −0.004 m and the absolute residual mean is 0.007 m; the root mean square is 0.008 m and normalized root mean square is 13.808%.

**Figure 4:**
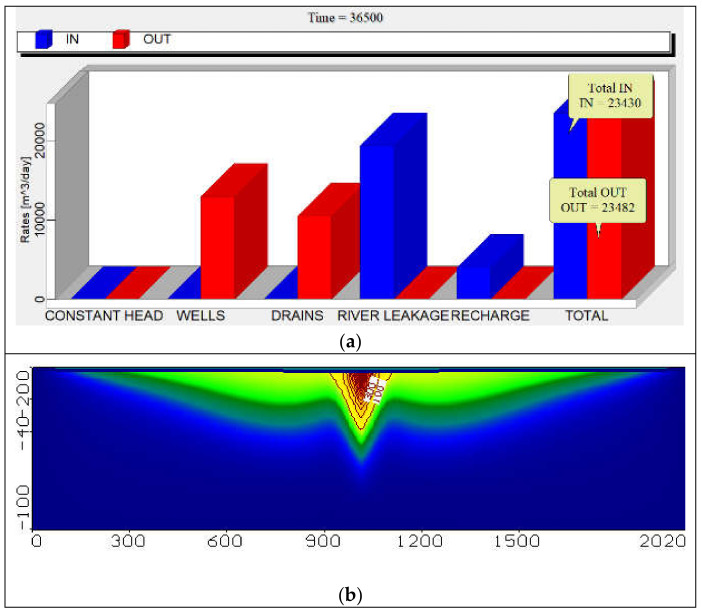
Zone budget and aerial view concentration of COD in the study area (Base case).

The zone budget is shown in Figure 4a where the total Inflow is 23,430 m^3^/day and Outflow is 23,482 m^3^/day and the difference (In-Out) is −52.01 m^3^/day. Figure 4b shows the distribution of concentration for COD in the study area without abstraction from the aquifer (base case). Where the extension at depth of screen at 25 m and 50 m reached 48.60, 19.41 ppm alternatively for selected observation well for contamination at distances X = 1500 m, Y = 100 m.

**Figure 5:**
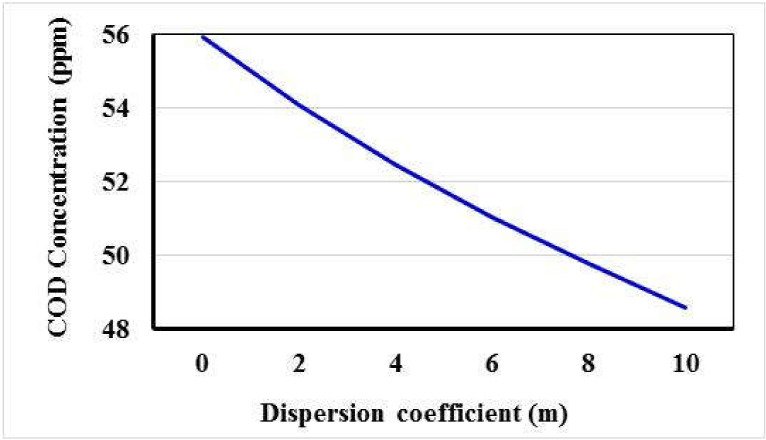
Relationship between COD concentration and dispersion coefficient.

Figure 5 presents the concentration for COD with dispersion coefficient for 0, 2, 4, 6, 8, and 10 m, the concentration reached 55.94, 54.09, 52.48, 51.06, 49.77, and 48.60 ppm. The results showed that the contamination has decreased with increasing the dispersion coefficient.

The results are accepted by results of other studies (Badaruddin et al. (2015) [30]. A number of scenarios were performed to investigate the effect of using untreated wastewater in irrigation, including four scenarios by increasing abstraction rates (scenario 1), increasing recharge rates (scenario 2), combination between these scenarios (scenario 3), and mixing untreated wastewater with treated wastewater (scenario 4) which are presented in Table 2, also the different values of pumping rate (*Q_pump_*), recharge (*R*), combination, and mixing are presented in Table 2. The management scenario is necessary for the protection of freshwater body in arid and semi-arid regions where the water resources are limited, and the groundwater is the main source for water supply in these areas. Therefore, this required to mixing the freshwater by treatment with wastewater using different percentage of mixing.

## 3. Results

The different scenarios of untreated wastewater and freshwater were considered in the current study including four scenarios, the first is pumping rate (*Q_pump_*), the second is wastewater recharge rates (*R*), the third is combination, and the fourth is mixing untreated wastewater with freshwater (treated wastewater).

### 3.1. Investigation of Groundwater Contamination

#### 3.1.1. Effect of Increasing Pumping Rates

**Figure 6:**
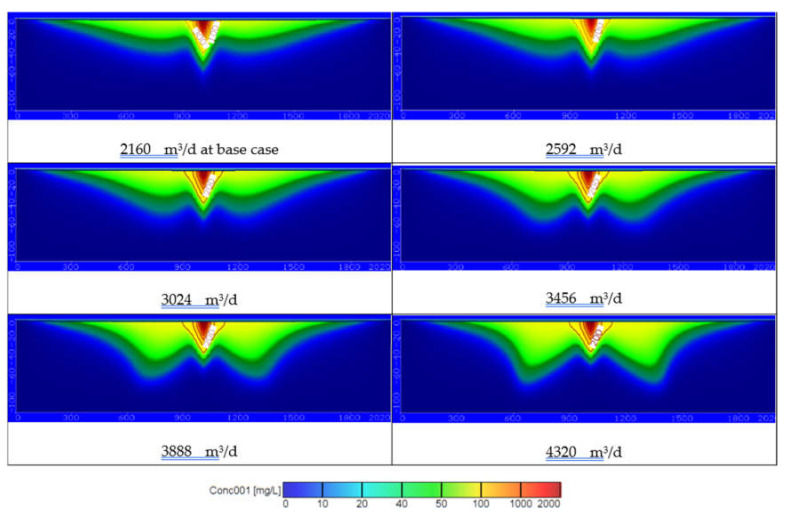
The distribution of COD in groundwater contamination for different pumping rates.

In this scenario, the impact of over pumping rates (*Q_pump_*) on groundwater quality are studied using five values of 2592, 3024, 3456, 3888, and 4320 m^3^/d due to over population and shortage in freshwater resources, while the recharge and the wastewater concentration were remained constant by 365 mm/year and 97.4 ppm, respectively. Figure 6 shows the distribution of contaminant COD through the study area. The results showed that over pumping rates led to increase the distribution of COD in the groundwater and the concentration reached 52.37, 57.29, 62.52, 68.42, and 74.49 ppm at depth 25 m and reached 21.5, 24.14, 26.66, 30.73, and 38.13 ppm at a depth of 50 m for the different pumping rates (see Figures 10a and 11). This stage indicated that over pumping must be optimized to protect groundwater quality for agriculture and drinking water.

#### 3.1.2. Effect of Increasing Wastewater Recharge Rates

**Figure 7:**
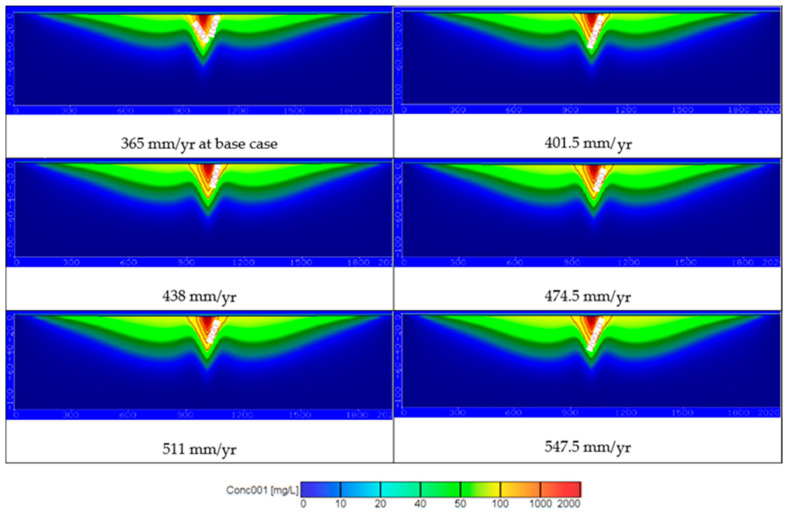
The distribution of COD in groundwater contamination for different wastewater recharge rates.

In this scenario, the effect of increasing wastewater recharge rates (*R*) on the contamination of the groundwater due to the shortage of surface water. Five values of recharge rates are examined including 401.5, 438, 474.5, 511, and 547.5 mm/year while pumping rate and the wastewater concentration were remained constant at 2160 m^3^/d and 97.4 ppm, respectively. The distribution of contaminates through the current study area is presented in Figure 7. The contamination reached to 52.73, 56.02, 59.29, 62.8, and 65.65 ppm at depth 25 m and 22.27, 24.6, 27.53, 30.73, and 32.7 ppm at a depth of 50 m (see Figures 10b and 11). The results of this scenario illustrated that with high rates of wastewater recharge rates led to increase the distribution of COD in groundwater contamination; moreover, using large quantities of untreated wastewater in agricultural must be managed to protect the groundwater contamination.

#### 3.1.3. Effect of Combination

**Figure 8:**
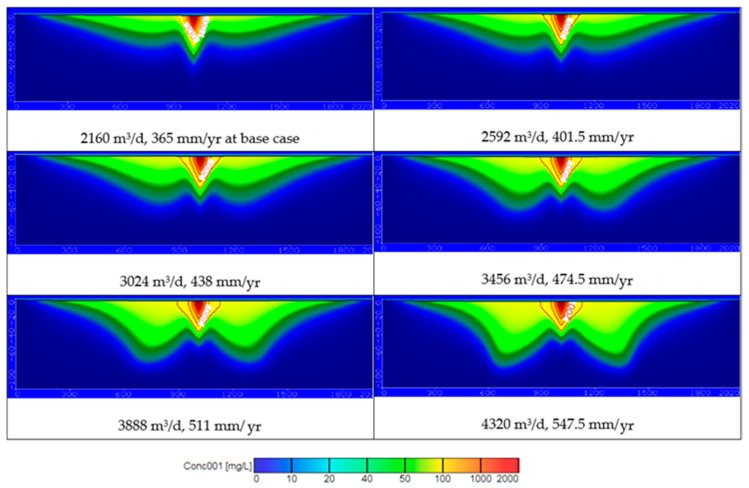
The distribution of COD in groundwater contamination for combination of different pumping and wastewater recharge rates.

In this scenario, in the combination case the pumping rates increased by 20, 40, 60, 80, and 100% to be 2592, 3024, 3456, 3888, and 4320 m^3^/d while the untreated wastewater recharge rates were increased by 10, 20, 30, 40, and 50% to be 401.5, 438, 474.5, 511, and 547.5 mm/year at concentration COD of 97.4 ppm. The contamination results for groundwater reached 56.60, 65.53, 72.55, 79.48, and 85.70 ppm at a depth of 25 m and reached 24.79, 30.59, 36.51, 44.54, and 56.06 ppm at a depth of 50 m. The distribution of the contamination is shown in Figure 8. Additionally, increase of the abstraction with untreated wastewater recharge rates contribute the increase of aquifer contamination (see Figures 10c and 11).

### 3.2. Protection of Groundwater from Using Untreated Wastewater

In this scenario, management of groundwater contamination from using the untreated wastewater in agricultural is applied by mixing it with freshwater. The untreated wastewater recharge rates are decreased by 10, 20, 30, 40, and 50% to reach 492.75, 438, 383.25, 328.5, and 273.75 mm/year with constant concentration of 97.4 ppm, while the freshwater recharge rates are increased to reach 54.75, 109.5, 164.25, 219, and 273.75 mm/year with concentration of 52.98 ppm [19], where the sum of the total recharge is constant 547.5 mm/year and the pumping rates were remained constant 4320 m^3^/d. The change in concentration reached 93, 88.52, 84.14, 79.7, and 75.19 ppm by mixing with freshwater by 10, 20, 30, 40, and 50% from untreated wastewater. Using the principle of salt mass balance to estimate the concentration of irrigation water after mixing with freshwater and the equation is as following [31]:

(Mass flow rate of pollutants) in = (Mass flow rate of pollutants) out

(4) $$Q_{W}\times C_{w}\;+Q_{us}\times C_{us}\;=\;Q_{ds}\times C_{ds}$$

where: *Q_w_*: Wastewater flow rate (m^3^/d), *C_w_*: concentration of a pollutant (ppm), *Q_us_*: stream flow rate (m^3^/d), *C_us_*: concentration of the pollutant (ppm), *Q_ds_*: downstream flow rate m^3^/d, and *C_ds_*: concentration of the mix after discharge (ppm).

**Figure 9:**
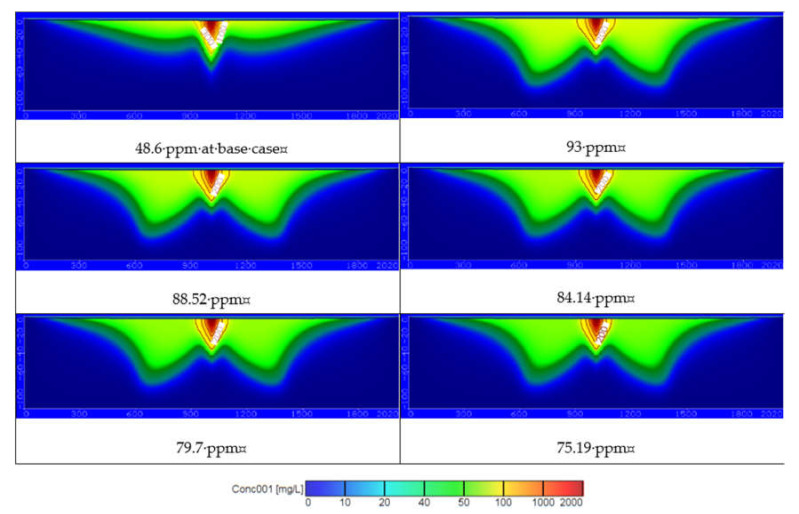
The distribution of COD in groundwater contamination for using different rates of treated wastewater recharge rates.

**Figure 10:**
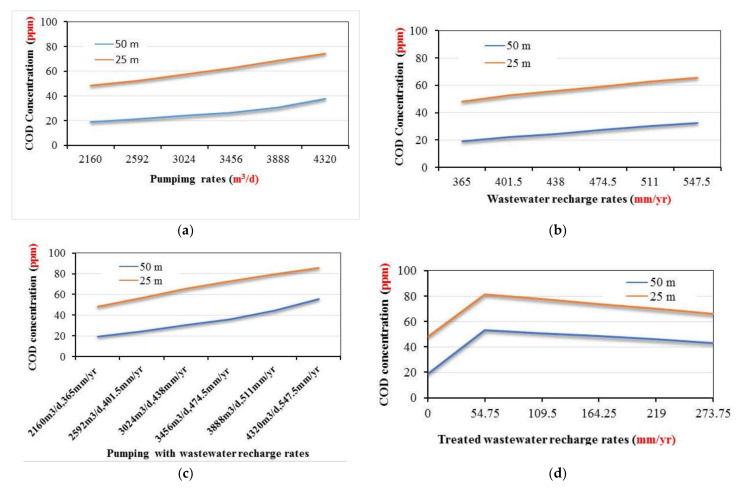
Relation between COD concentrations at different depths and (**a**) pumping rates, (**b**) recharge rates, (**c**) pumping with recharge rates, and (**d**) treated wastewater recharge rates.

**Figure 11:**
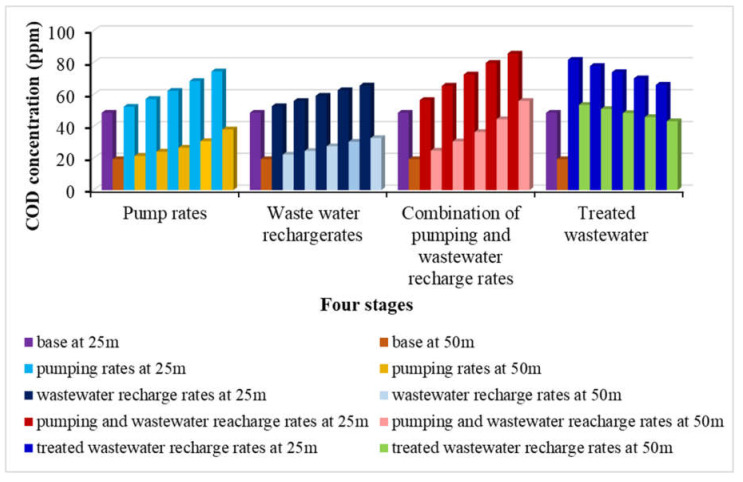
Comparison between the proposed scenarios 1, 2, 3 and 4 for COD concentrations.

Figure 9 shows the distribution of contaminate through this study area for different values of COD and the contamination reached 81.82, 77.88, 74.03, 70.12, and 66.15 ppm at a depth of 25 m and 53.53, 50.95, 48.43, 45.87, and 43.28 ppm at a depth of 50 m. Figure 10d and Figure 11 show that decreasing of wastewater concentration with using the freshwater by treated wastewater led to decrease the distribution of COD in groundwater. Construction of wastewater treatment plants must be extended to decrease the use of untreated wastewater and increase the freshwater to improve the irrigation and drinking water quality and protect the groundwater aquifer from contamination.

## 4. Discussion

Using untreated and treated wastewater in arid and semi-arid regions is considered an attempt to overcome the shortage of water resources in these areas. Results of current study for the COD concentration at 25 and 50 m depths for different scenario are summarized in Table 3. Figure 11 shows the distribution of COD concentration in the aquifer, which reached 52.37, 57.29, 62.52, 68.42, and 74.49 ppm at a depth of 25 m and reached 21.5, 24.14, 26.66, 30.73, and 38.13 ppm at a depth of 50 m by using different pumping rates (*Q_pump_*) of 2592, 3024, 3456, 3888, and 4320 m^3^/d compared with 2160 m^3^/d at base case. The results are consistent with Abd-Elhamid et al. [19] who approved that increasing pumping rates led to increase the aquifer contamination. Additionally, Figure 11 shows the increasing of the wastewater recharge rates (*R*) to 401.5, 438, 474.5, 511, and 547.5 mm/year compare with 365 mm/year at base case led to increase the COD distribution in aquifer to 52.73, 56.02, 59.29, 62.8, and 65.65 ppm at a depth of 25 m and 22.27, 24.6, 27.53, 30.73, and 32.7 ppm at a depth of 50 m. These findings according to Abd-Elaty et al. [21] who studied the effect of increasing fertilizers concentration and combination of pumping rates with fertilizer rates led to increase aquifer contamination. Applying different pumping rates with increasing wastewater recharge rates as in the combination case increased COD in groundwater to 56.60, 65.53, 72.55, 79.48, and 85.70 ppm at a depth of 25 m and reached 24.79, 30.59, 36.51, 44.54, and 56.06 ppm at a depth of 50 m as shown in Figure 11. Finally, as an attempt to protect the groundwater from using untreated wastewater in irrigation, it was mixed with treated wastewater to reduce the concentration of COD to 93, 88.52, 84.14, 79.7, and 75.19 ppm. The distribution of groundwater contaminant reached 81.82, 77.88, 74.03, 70.12, and 66.15 ppm at a depth of 25 m and 53.53, 50.95, 48.43, 45.87, and 43.28 ppm at a depth of 50 m shown in Figure 11. This agrees with Abd-Elaty et al. [23] who studied the mitigation of polluted stream by mixing with treated domestic and industrial wastewater can decrease the polluted load in the streams, which could be used in irrigation and protect the ground water quality.

Figure 10a and Figure 11 show the results through a liner relation between increasing pumping rates and the concentration of COD at two depths at 25 and 50 m. The pumping rates ranged between 2160–4320 m^3^/d and the COD concentration ranged between 52.37–74.49 ppm for a depth of 25 m and decreased for a depth of 50 m to range between 21.5–38.13 ppm. Additionally, Figure 10b and Figure 11 show the relation between wastewater recharge rates and the concentration of COD for two depths at 25 and 50 m in a linear relation. Using wastewater recharge rates ranged between 365–547.5 mm/year increases the COD concentration from 52.73 to 65.65 at a depth of 25 m and decreased for a depth of 50 m to range between 22.27–32.7 ppm. Figure 10c and Figure 11 show the combination between increasing pumping rates and wastewater recharge rates and the COD concentration. The combination of pumping rates ranged between 2160–4320 m^3^/d and wastewater recharge rate with range between 365 and 547.5 mm/year increase the COD concentration from 56.60 to 85.70 ppm at a 25 m depth and from 27.79 to 56.06 ppm at a depth of 50 m. In Figure 10d and Figure 11 the relation between the treated wastewater recharge rates and the COD concentration for two depths of 25 and 50 m. Mixing of treated wastewater recharge rate with range from 54.75 to 273.75 mm/year decreased the COD concentration from 81.82 to 66.15 ppm at a depth of 25 m and for a depth of 50 m range from 53.53 to 43.28 mm/year.

Groundwater quality is defined as a combination of human and natural influences, and one quality issue is monitored, such as saltwater intrusion, industrial spillage, agricultural pesticides, etc. Among the most important quality issues worldwide are the physical components (filtering and dispersion) and biochemical (cell synthesis) and geochemical (oxidation–reduction, adsorption–desorption, and Ionic strength) [32].

Recharging the aquifer with water that has been treated and recycled will become among the basic factors for strategic plans for water management, and this is a result of water shortage. The existence of the pollutant inside the aquifer has a long-term impact after the withdrawal; complementary treatment materials should not be added due to recharge, which may be important to meet the standards. In order to make available quantities of water resources and to develop the recharge of the aquifer, a distinction must be made between aquifers that are prepared for drinking and those not prepared for drinking [33]. Measures to protect groundwater sources must be anticipated and include the following.

Preserving wetlands as they purify water, reducing the use of pesticides and chemicals, paying attention to remediate dumping and spills, the appropriate design of landfills and following up on their maintenance, selecting a suitable site for wastewater treatment systems, follow up the maintenance of the petroleum storage sector, attempt to prevent or stop underground injection, and trying to control pollution resulting from leaking rainwater [33].

## 5. Conclusions

Shortage of freshwater supplies has contributed to the use of unconventional water resources in scarcity regions including untreated wastewater without control in agriculture. This will affect groundwater quality. The current study was carried out using the numerical model of MT3D to investigate the impact of using raw wastewater in irrigation under increasing the abstraction rates (scenario 1), wastewater concentration (scenario 2), combination between (scenario 1, 2, and 3); additional management scenarios were applied by mixing untreated wastewater with freshwater. The model was simulated using a square hypothetical case of length and width equal 2000 m with 100 m in depth. This case study is anisotropic and homogeneous. The result of the first scenario by increasing the abstraction rates by 2592, 3024, 3456, 3888, and 4320 m^3^/d increased the contaminant to 52.37, 57.29, 62.52, 68.42, and 74.49 ppm at a depth of 25 m and 21.5, 24.14, 26.66, 30.73, and 38.13 ppm at a depth of 50 m from the base case at 48.60 and 19.41 ppm at depths of 25 and 50 m, respectively. The result revealed that using untreated wastewater in irrigation must be controlled to protect groundwater quality in aquifer.

For second scenario, the results showed that increasing the wastewater recharge rates led to increase the contamination to 52.73, 56.02, 59.29, 62.80, and 65.65 ppm at a depth of 25 m and 22.27, 24.60, 27.53, 30.37, and 32.70 ppm at a depth of 50 m at recharge rates of 401.5, 438, 474.5, 511, and 547.5 mm/year.

The groundwater recharge by wastewater should be managed to protect the groundwater from contamination. In the third stage combination of increasing the abstraction, wastewater recharge rates increased the contamination to 56.60, 65.53, 72.55, 79.48, and 85.70 ppm at a depth of 25 m and 24.79, 30.59, 36.51, 44.45, and 56.06 ppm at a depth of 50 m from the base case at 48.60 and 19.41 ppm at depths of 25 and 50 m, respectively. Finally, the scenario of protecting groundwater by mixing untreated wastewater with treated wastewater to manage the contamination, where the contamination reached 81.82, 77.88, 74.03, 70.12, and 66.15 ppm at a depth of 25 m and 53.53, 50.95, 48.43, 45.87, and 43.28 ppm at a depth of 50 m from the base case at mixing values of 93, 88.52, 84.14, 79.7, and 75.19 ppm. This scenario is the best for groundwater protection from using untreated wastewater in agricultural that could be applied in these regions.

## Figures and Tables

**Table 1:** Hydraulic Parameters for the hypothetical case study.

Parameter	Hydraulic Conductivity		*S_s_* 1/m	*S_y_* -	*n_eff_* -	*n_total_* -
	*K*_x_ = *K*_y_ (Horizontal) m/day	*K*_z_ (Vertical) m/day				
Value	5	0.5	27 × 10^−7^	0.2	20%	35%

**Table 2:** Proposed scenarios for untreated wastewater with mixing by treated wastewater.

	Stage Type	Scenario No.	*Q*_pump_ m^3^/d	*R* mm/year		*C* ppm			% of Mixing
				Untreated Wastewater	Treated Wastewater	Untreated Wastewater	Treated Wastewater	Mix	
	Base	1	2160	365	0	97.40	0	0	0
Investigation (I)	(I_1_) Aquifer pumping rates (*Q*_pump_)	2	2592	365	0	97.40	0	0	0
		3	3024						
		4	3456						
		5	3888						
		6	4320						
	(I_2_) Untreated wastewater (*R*)	7	2160	401.50	0	97.40	0	0	0
		8		438					
		9		474.50					
		10		511					
		11		547.50					
	(I_3_) Combination	12	2592	401.50	0	97.40	0	0	0
		13	3024	438					
		14	3456	474.50					
		15	3888	511					
		16	4320	547.50					
Management (M)	(M_1_) Mixing	17	4320	492.75	54.75	97.40	52.98	93	61.80
		18		438	109.50			88.52	58.86
		19		383.25	164.25			84.14	55.95
		20		328.50	219			79.70	52.99
		21		273.75	273.75			75.19	50

**Table 3:** Results of COD concentration at 25 and 50 m depths for different scenario.

Stage Type	Scenario No	COD Concentration in ppm at 25 m	COD Concentration in ppm at 50 m
Base case	1	48.6	19.41
Aquifer pumping rate (*Q_pump_*)	2	52.37	21.5
	3	57.29	24.14
	4	62.52	26.66
	5	68.42	30.73
	6	74.49	38.13
Untreated wastewater (*R*)	7	52.73	22.27
	8	56.02	24.6
	9	59.29	27.53
	10	62.8	30.37
	11	65.65	32.7
combination	12	56.60	27.79
	13	65.53	30.59
	14	72.55	36.51
	15	79.48	44.54
	16	85.70	56.06
mixing	17	81.82	53.53
	18	77.88	50.95
	19	74.03	48.43
	20	70.12	45.87
	21	66.15	43.28

## Data Availability

Not applicable.
